# Meta-Analysis of Randomized Controlled Trials Comparing Latanoprost with Timolol in the Treatment of Asian Populations with Chronic Angle-Closure Glaucoma

**DOI:** 10.1371/journal.pone.0096852

**Published:** 2014-05-09

**Authors:** Shi-Ming Li, Ru Chen, Yuan Li, Zhi-Rong Yang, Qiu-Ju Deng, Zheng Zhong, Moh-Lim Ong, Si-Yan Zhan

**Affiliations:** 1 Beijing Tongren Eye Center, Beijing Tongren Hospital, Capital Medical University, Beijing, China; 2 Department of Epidemiology and Health Statistics, Peking University School of Public Health, Beijing, China; 3 Pfizer China, Beijing, China; 4 Department of Genetics, Peking University School of Oncology, Beijing Cancer Hospital & Institute, Hai Dian District, Beijing, China; 5 Pfizer Global Pharmaceuticals, New York, New York, United States of America; Bascom Palmer Eye Institute, University of Miami School of Medicine, United States of America

## Abstract

**Background:**

To evaluate the efficacy and safety of latanoprost compared with timolol in the treatment of Asian patients with chronic angle-closure glaucoma (CACG).

**Methods:**

Relevant trials were identified through systematic searches of Medline, EMBASE, PubMed, Cochrane Library, Google Scholar and several Chinese databases. The main outcome measures included absolute and relative reduction of intraocular pressure (IOP) at mean, peak and trough from baseline, ocular adverse effects and systemic adverse events.

**Results:**

Seven randomized controlled trials with 685 patients were included. In comparison with timolol, latanoprost reduced absolute IOP in CACG patients by more than 2.3 mmHg (95%CI, 1.8∼2.9, P<0.01), 2.4 mmHg (95%CI, 1.9∼2.9, P<0.01) and 2.5 mmHg (95%CI, 1.6∼3.3, P<0.01) at mean, peak and trough, respectively. As for relative IOP, there is 9.0% (95%CI, 6.6∼11.4, P<0.01), 9.7% (95%CI, 7.6∼11.8, P<0.01), and 10.8% (95%CI, 7.4∼14.3, P<0.01) greater reduction among latanoprost users than among timolol users. The differences were statistically significant at all time points (1, 2, 4, 8, 12, and 24 weeks). More ocular adverse effects (OR = 1.49, 95% CI, 1.05∼2.10, P = 0.02) and less systemic adverse events (OR = 0.46, 95% CI, 0.25∼0.84, P = 0.01) were observed in latanoprost group in comparison with timolol group.

**Conclusion:**

Compared with timolol, latanoprost was significantly more effective in lowering IOP of Asian patients with CACG, with higher risk of ocular adverse effects but lower risk of systemic adverse events, and might be a good substitute for CACG patients.

## Introduction

Chronic angle-closure glaucoma (CACG), a disease characterized by elevated intraocular pressure (IOP) resulting from gradual angle closure and a decrease in aqueous humor outflow, is considered as a major form of glaucoma in Asia [1]–[6]. First-line therapy for CACG is peripheral iridotomy (PI) which is an invasive procedure [7], [8]. However, for many CACG patients, PI alone is insufficient to control IOP. Topical β-blockers such as timolol which reduces the aqueous humor generation is usually added to these patients to further lower their IOP.

Latanoprost, a representative of prostaglandin analogs which can significantly reduce IOP by increasing uveoscleral outflow, has been proved to be more effective in lowering IOP in patients with primary open-angle glaucoma (POAG) or ocular hypertension (OH) than timolol [9], [10]. As for CACG patients, however, the differences between these two eyedrops in efficacy and safety have not been systematically evaluated. Previous studies [11], [12] were designed differently, and were mainly performed in Asia-Pacific population. Whether there is difference in lowering IOP among different populations remains unclear. In addition, how long latanoprost can keep a stable level in lowering IOP than timolol is also of concern.

The purposes of this meta-analysis was to systematically evaluate the efficacy and safety of latanoprost compared with timolol in treating Asian patients with CACG, to compare the efficacy and safety difference between these two drugs in Chinese Mainland population and other Asia-Pacific population, and to evaluate how long could this difference last and the robustness of the available evidence.

## Methods

### Search strategy

Articles were identified through a computerized search up to March 2013 in the following data sources: MEDLINE, EMBASE, PubMed and Cochrane Library, Google Scholar and several Chinese databases including CBM (Chinese Biomedical Literature Database), CNKI (China National Knowledge Infrastructure), WANFANG DATA and VIP Database. The keywords were *angle-closure glaucoma, latanoprost, xalatan, timolol and timoptol*. Mesh terms were used if available. Otherwise the keywords were searched in full text. See Table S1 for search strategy in PubMed. References within the retrieved articles from the above search were used to search for additional trials. We also searched in Chinese Clinical Trial Registry, WHO International Clinical Trials Registry Platform and ClinicalTrials.gov for ongoing trials.

### Inclusion criteria

Relevant clinical trials (RCT) were selected based on the protocol-determined selection criteria. (i) Study type: RCTs. (ii) Population: Patients with chronic angle-closure glaucoma, including primary chronic angle-closure glaucoma and residual chronic angle-closure glaucoma after laser or surgical treatment. (iii) Intervention: Latanoprost versus timolol in each group without combination of any other drugs. (iv) Primary outcome measures: Absolute and relative IOP reduction from baseline at mean, peak and trough were used for efficacy analysis; Occurrences of ocular and systemic adverse effects were used for safety analysis.

For latanoprost 0.005% once daily, the time points were as close to 12 hours for peak and 24 hours for trough after administration as possible. And for timolol 0.5% twice daily, the time points were as close to 2 hours for peak and 12 hours for trough after administration as possible [13]. Mean IOP was defined as the mean value of measurements at all time points throughout the 24-h cycle.

A broad focus on adverse effects was chosen to detect a variety of adverse effects, whether known or previously unrecognized. All cases reported in the studies with information falling under any of the terms ‘adverse effect’, ‘adverse drug reaction’, ‘side effect’, ‘toxic effect’, ‘adverse event’ and ‘complication’ were considered as adverse effects. Ocular adverse effects were defined as adverse effects related with eyes and the remaining was classified as systemic adverse effects in accordance with the term used in the trials.

### Screening and data extraction

Trial eligibility was determined by two authors independently (C.R., D.Q.J.). Title, abstract, and medical subject heading words of the obtained publications were initially used for a rough judgment on eligibility of an article. Of the remaining identified publications, the full texts were downloaded for further judgment. Data extraction was performed according to a customized form by two authors (C.R., D.Q.J.) independently. The form covered information on article characteristics (authors, year of publication, location, language, center and funding), study design (type of study, control and intervention, length of wash-out period and study), participants (number, age, gender, race and surgical treatment), and outcomes (IOP, ocular and systemic adverse effects). Any disagreements in article selection and data extraction were resolved by discussion or a third authors (Y.Z.R).

### Quality assessment

Methodological quality was evaluated (in duplicate by C.R. and D.Q.J.) using the Cochrane Collaboration tool for assessing risk of bias of clinical trials and figures were generated using RevMan (5.2) [14]. The tool included six individual domains: sequence generation, allocation concealment, blinding, incomplete outcome data, selective outcome report and other sources of bias. Each domain had one entry with the judgment of ‘Low risk’, ‘High risk’, or ‘Unclear risk’ of bias and a description of the design, conduct or observations that underlie the judgment. We contacted with the authors by email and waited for a response for at least 4 weeks when information reported in the trials was insufficient to make a judgment.

### Statistical analysis

Outcome measures were assessed on an intention-to-treat (ITT) analysis. The ITT population was comprised of all randomized patients who received a minimum of 1 dose of active treatment and provided a valid baseline measurement. Otherwise, available case analysis was used.

Original data were obtained from the articles as much as possible; data that could not be obtained were calculated when necessary. When mean and standard deviation (SD) of IOP reduction (IOPR) were not available directly, they were calculated by the formulas as the following [15]–[17]: 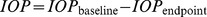


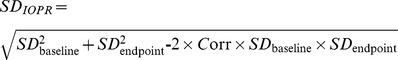


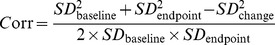



The correlation coefficient indicates correlation between baseline SD and endpoint SD of the measurement, and was calculated from trials with known SD_change_. For studies that only reported standard errors (SEs), SD was calculated by the formula 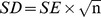. The mean and SD of relative IOPR (IOPR%) were then estimated by the formulas [13], [18], [19]: 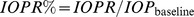


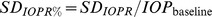



A random-effects model was used if trials were heterogeneous on the basis of the Q statistic for heterogeneity and the reasons for the heterogeneity could not be identified [20], [21]. Otherwise a fixed-effect model was used to estimate the pooled effects.

The mean difference (MD) was used to measure the absolute difference between the mean values of two groups in a clinical trial. The MD here referred to the subtraction of mean value in latanoprost group from the mean value in timolol group. The odds ratio (OR) were estimated for the adverse effects using ITT analysis. In the pooling of odds ratio, the Mantel-Haenszel method was used for the fixed-effect model. The method described by DerSimonian and Laird [22] was used for the random-effects model.

Subgroup analyses between Chinese Mainland population and other Asia-Pacific population were used to investigate heterogeneity of efficacy in different populations. Sensitivity analyses were undertaken to evaluate the effect of quality of randomized controlled trials in terms of the study design, withdrawal rate and pharmaceutical industry support. To detect publication biases, we explored asymmetry in funnel plots. Egger's measure of publication bias was calculated if more than 10 studies were included [23].

## Results

### Study eligibility

**Figure 1:**
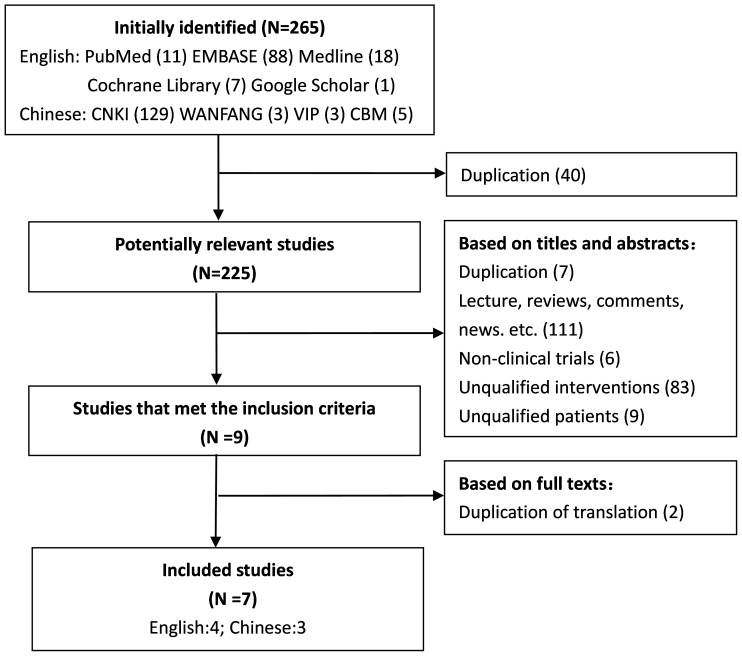
Flow diagram of the results of the search strategy.

The selection flow of studies was summarized in Figure 1. In case more than one reason for exclusion was present, only the first reason encountered was listed. The initial search identified 125 studies in English and 140 studies in Chinese. Finally, 7 RCTs [24]–[30] were included in this meta-analysis.

### Trials characteristics

Characteristics of 7 RCTs were summarized in Table 1. These RCTs were conducted in various countries including China [26], [28]–[30], Indonesia [25], Malaysia [25], Philippines [25], Singapore [24], [25], Thailand [25], and India [27]. A total of 685 patients were included with 343 in latanoprost group and 342 in timolol group. Three trials [24], [27], [30] claimed that the authors had no relevant financial interest. Baseline and endpoint IOP were summarized in Table 2. Four trials [24], [25], [27], [28] reported the severity of the adverse effects and no treatment related serious adverse events were observed.

**Table 1:** Baseline Characteristics of Eligible Randomized Clinical Trial.

Studies	Design	Latanoprost (%)	Timolol(%)	Center	Location	Funding	Total No.	Withdrawals (%)	Age (Mean±SD)	Sex (M/F)	Population	Types of glaucoma	Operations	Washout period	Length
Aung 2000	DB, PG	0.005 eve	0.5bid	Multi	Singapore	Singapore Eye Research Institute (partly)	32	9.4	T:64±7 C:64±8	16/16	Chinese, Malay, Indian	Primary chronic angle-closure glaucoma	After peripheral iridotomy	Yes	2 w
Chew 2004	DB, PG	0.005 eve	0.5bid	Multi	Hong Kong, Indonesia, Malaysia, Philippines, Singapore, Taiwan, Thailand	Pharmacia Corporation	275	6.2	T:63.3±9.4 C:63.1±9.5	70/205	Asian or Pacific Islander	Chronic angle-closure glaucoma	After peripheral iridotomy	Yes	12 w
Kong 2005	SB, PG	0.005 eve	0.5bid	Single	China	NR	49	16.3	T:61.6±8.8 C:60.4±7.9	14/35	Chinese	Residual angle-closure glaucoma	After out-filtrating surgery or iridotomy	Yes	8 w
Liu 2006	NR, PG	0.005 eve	0.5bid	Single	China	NR	60	0	-	35/25	Chinese	Chronic angle-closure glaucoma	NR	Yes	2 w
Sihota 2004	DB, CR	0.005 eve	0.5bid	Single	India	NR	30	0	57.7±7.4	18/12	Indian	Primary chronic angle-closure glaucoma	After laser iridotomy	Yes	3 m
Wang 2002	NR, PG	0.005 eve	0.5bid	Single	China	NR	68	17.6	-	-	Chinese	Residual angle-closure glaucoma	After peripheral iridotomy	Yes	6 m
Zhao 2012	OL, PG	0.005 eve	0.5bid	Multi	China	Pharmacia Upjohn, China (acquired by Pfizer Inc)	142	0.7	T:63±7.3 C:61.3±8.9	47/94	Chinese	Chronic angle-closure glaucoma	After laser or surgical peripheral iridotomy	Yes	8 w

CR indicates crossover; DB, double blind; NR, not reported; OL, open label; PG, parallel group; SB, single blind.

eve =  evening regimen, bid =  twice per day, w = week, m =  month.

**Table 2:** Outcome Measurements of Eligible Randomized Clinical Trial.

Studies	Time Points	Baseline IOP (mmHg) (Mean(SD))						Endpoint IOP (mmHg) (Mean(SD))						Ocular adverse effects (Times)		Systemic adverse effects (Times)	
		Mean		Peak		Trough		Mean		Peak		Trough					
		Latanoprost	Timolol	Latanoprost	Timolol	Latanoprost	Timolol	Latanoprost	Timolol	Latanoprost	Timolol	Latanoprost	Timolol	Latanoprost	Timolol	Latanoprost	Timolol
Aung2000	Mean,peak(9AM), trough (5PM)	25.7(3.6)	25.2(4.1)	27.2(3.8)	26.8(4.5)	24.2(3.8)	23.5(4.3)	16.9(5.2)	19.4(2.4)	17.0(4.7)	20.0(2.6)	16.8(5.8)	18.9(2.8)	14	12	0	1
Chew2004	Mean,peak(9AM), trough(5 PM)	25.0(5.5)	25.9(6.3)	25.2(5.5)	25.9(6.5)	24.8(6.0)	25.9(7.1)	17.5(5.0)	20.7(6.9)	NR	NR	NR	NR	61	57	17	28
Kong2005	Mean,peak(9AM), trough(4PM)	24.8(3.3)*	25.8(3.9)*	24.7(3.9)	26.0(4.4)	24.8(3.5)	25.5(4.2)	15.7(3.2)*	18.1(3.6)*	16.1(3.9)	17.5(4.0)	15.3(3.2)	18.8(4.1)	0	3	0	2
Liu2006	Peak(9AM)	NR	NR	25.3(4.1)	24.2(3.5)	NR	NR	NR	NR	19.1(3.4)	20.3(2.5)	NR	NR	2	0	0	0
Sihota2004	Mean,peak(10AM),trough(7PM)	23.4(2.1)	23.4(2.1)	24.6(3.9)	24.6(3.9)	22.4(3.1)	22.4(3.1)	15.3(1.8)	17.4(1.7)	14.6(2.8)	17.9(3.6)	15.6(3.1)	16.9(3.8)	6	2	0	0
Wang2002	Peak(9AM)	NR	NR	24.1(1.0)	24.1(1.1)	NR	NR	NR	NR	17.0(1.0)	19.1(1.3)	NR	NR	4	5	0	0
Zhao2012	Mean,peak(9AM),trough(4PM)	24.3(3.0)	24.2(3.0)	NR	NR	NR	NR	17.6(3.8)	19.3(3.9)	NR	NR	NR	NR	42	24	1	5

*Pooled value of IOP measured at 10 AM. and 4 PM; NR, not reported.

### Trials quality

**Figure 2:**
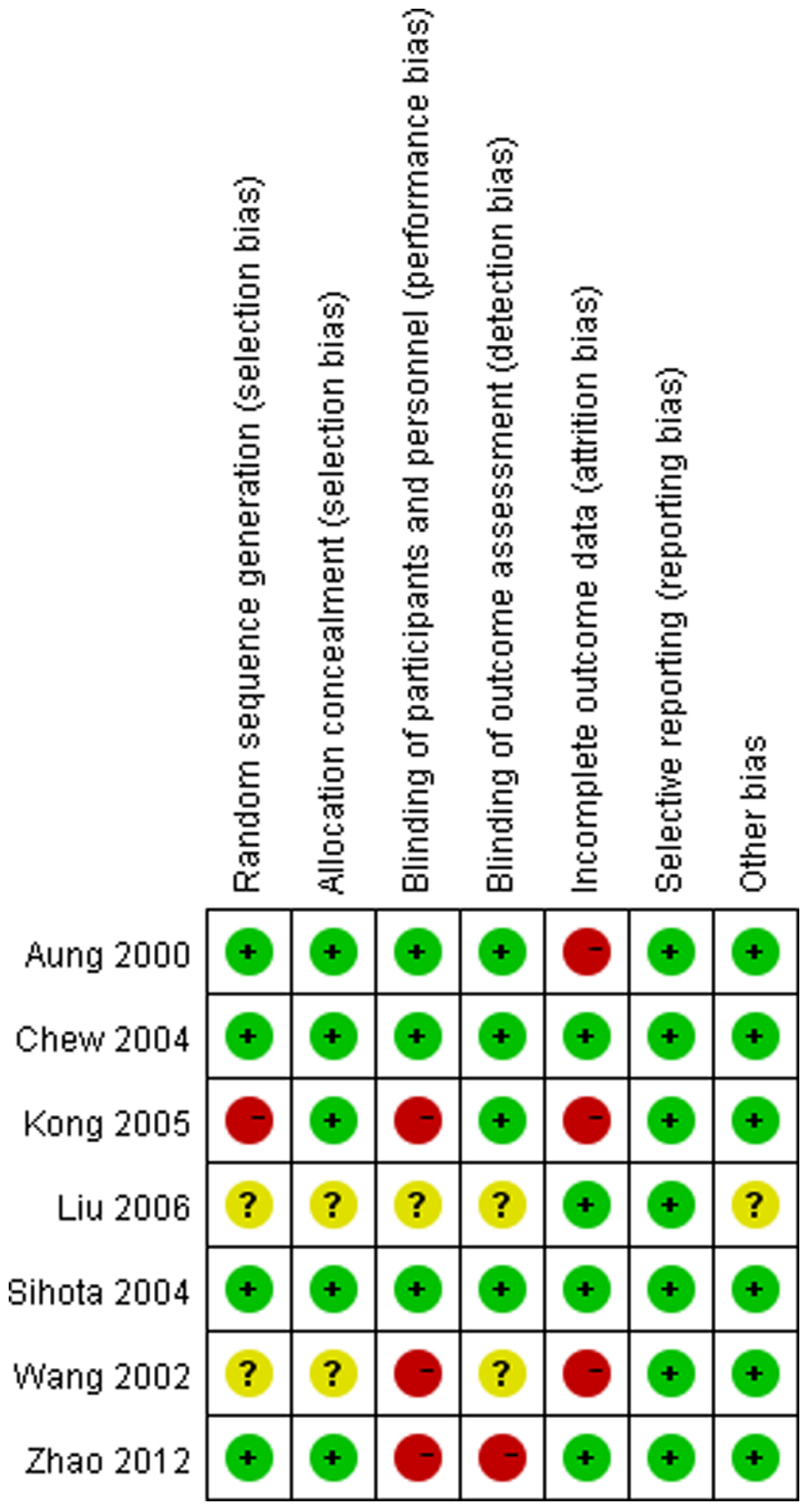
Risk of bias summary. Red stands for high risk of bias, green stands for low risk of bias and yellow stands for unclear risk of bias.

**Figure 3:**
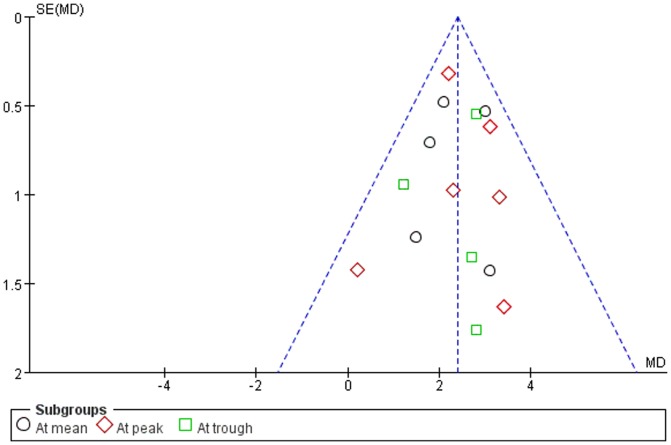
Funnel plots of RCTs comparing lantanoprost with timolol in IOP reduction.

Five authors [24], [25], [27], [28], [30] replied our emails with detailed information. Figure 2 presented all judgments (‘Low risk’, ‘High risk’ and ‘Unclear risk’) in a cross-tabulation of study by entry. We were confident of blinding of participants and personnel in three trials [24], [25], [27], blinding of outcome assessment in four trials [24], [25], [27], [28], random sequence generation in four trials [24], [25], [27], [30], and allocation concealment in five trials [24], [25], [27], [28], [30]. There was no clear evidence of publication bias on the funnel plot, although the number of publications included was small (Figure 3).

### Efficacy

**Figure 4:**
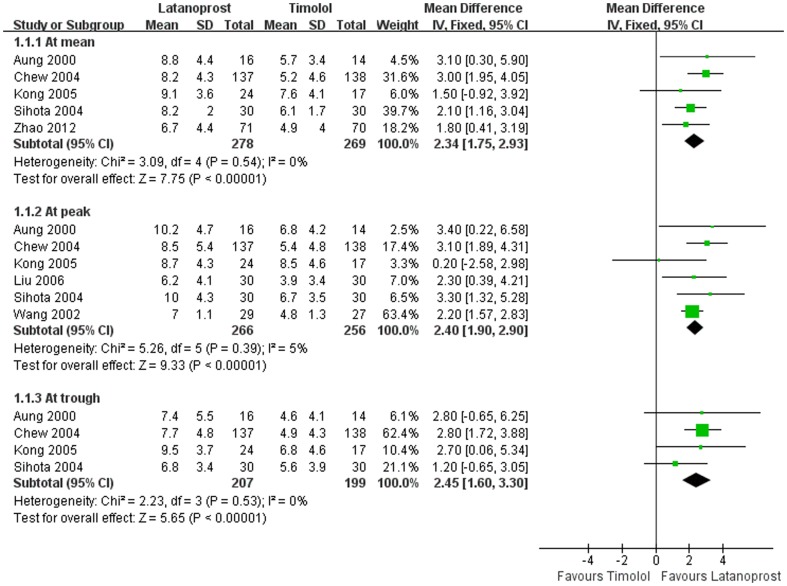
Comparison of Absolute IOP reductions between latanoprost and timolol.

**Figure 5:**
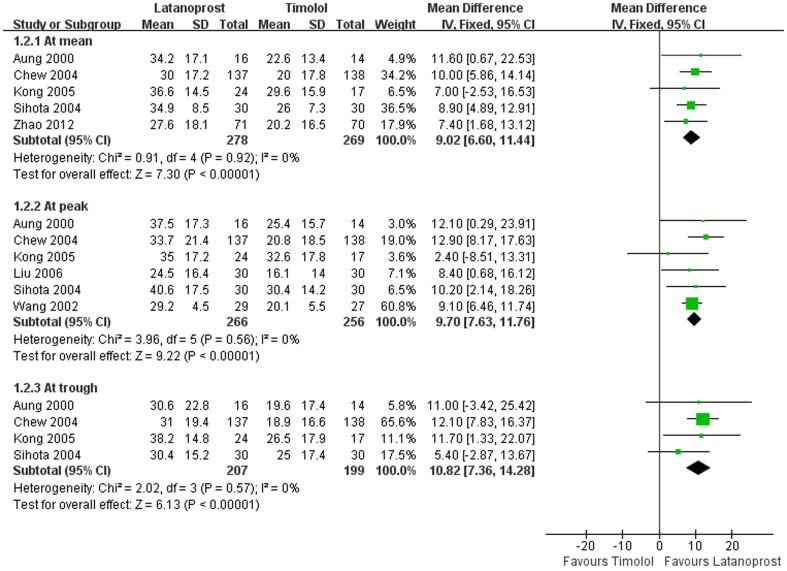
Comparison of Relative IOP reductions between latanoprost and timolol.

1. **Absolute IOP reduction.** Pooled absolute IOP reductions from baseline for latanoprost and timolol were shown in Figure 4. Mean differences in absolute IOP reduction between two groups were 2.3 mmHg (P<0.01) at mean, 2.4 mmHg (P<0.01) at peak and 2.5 mmHg (P<0.01) at trough, respectively.
2. **Relative IOP reduction.** Relative IOP reductions from baseline of two drugs were shown in Figure 5. Mean differences in relative IOP reduction between latanoprost and timolol were 9.0% (P<0.01) at mean, 9.7% (P<0.01) at peak, and 10.8% (P<0.01) at trough, respectively.
3. **Effects at different time points.** Absolute reductions in mean and peak IOP between latanoprost and timolol at various time points were shown in Table 3. The differences were all statistically significant with greater reduction in IOP of latanoprost.
4. **Sensitivity analysis.** All trials were divided into subgroups by method of blinding and withdrawal, respectively. There were no statistically significant differences in absolute and relative IOP reduction between double blind/single blind group and open label/not reported group (P = 0.26, 0.36), and between groups with withdrawal rate less than 10% or 10% or more (P = 0.10, 0.22). (See Table S2)
5. **Subgroup analysis.** Mean differences in absolute IOP reductions at average, peak and trough between latanoprost and timolol were 1.7 mmHg, 2.1 mmHg and 2.7 mmHg in Chinese Mainland population, and 2.5 mmHg, 3.2 mmHg and 2.4 mmHg in other Asia-Pacific population, respectively. For relative IOP reduction at mean, peak and trough, the mean differences between latanoprost and timolol were 7.3%, 8.7% and 11.7% in Chinese mainland population, and 9.6%, 12.2% and 10.7% in other Asia-Pacific population, respectively. The differences between subgroups were neither statistically significant in absolute nor relative mean IOP reduction (P>0.05). There were no significant differences in absolute IOP reduction between studies with pharmaceutical industry funding and studies without funding (P = 0.38), though greater mean difference of IOP reduction was observed in funded studies (2.6 mmHg, 95%CI, 1.7∼3.4) than the studies that did not report any funding (2.1 mmHg, 95%CI, 1.3∼3.0). (See Table S3 and Figure S1)

**Table 3:** Absolute IOP reduction from baseline at different time points.

Time Points	No. of trials	No. of patients		Mean difference (mmHg)(95%CI)	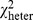 (p value)	Z_overall_ (p value)
		Latanoprost	Timolol			
IOP reduction at mean						
1 week	1	71	70	1.8(0.4,3.2)	-	2.54(0.01)
2 weeks	2	87	84	2.1(0.9,3.3)	0.58(0.45)	3.48(<0.01)
4 weeks	1	71	70	1.6(0.3,3.0)	-	2.32(0.02)
8 weeks	2	95	87	1.7(0.5,2.9)	0.04(0.83)	2.81(<0.01)
12 weeks	2	167	168	2.5(1.8, 3.2)	1.56(0.21)	6.99(<0.01)
IOP reduction at peak						
1 week	3	69	58	2.0(1.3,2.6)	0.99(0.61)	6.15(<0.01)
2 weeks	4	99	88	2.0(1.4,2.6)	1.79(0.62)	6.42(<0.01)
4 weeks	2	53	44	1.9(1.3,2.6)	1.00(0.32)	5.90(<0.01)
8 weeks	2	53	44	1.9(1.4,2.5)	1.54(0.21)	6.64(<0.01)
12 weeks	2	196	195	2.3(1.8,2.9)	3.45(0.18)	8.25(<0.01)
24 weeks	1	29	27	2.2(1.5,2.9)	-	6.29(<0.01)

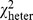
 = 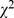 test for subgroup differences.

All pooling was undertaken using fixed effect model as no heterogeneity was detected by Q test.

### Safety

Overall, 129 ocular adverse events (AEs) and 18 systemic AEs were recorded in latanoprost group and 103 ocular AEs and 36 systemic AEs were recorded in timolol group by the 7 clinical studies in our analysis. The frequencies of the most common ocular and systemic AEs in both groups are shown in Table 4.

**Table 4:** Risk of adverse effects with latanoprost and timolol.

Adverse Effects	No. of trials	No. of events/No. of patients		Pooled OR (95%CI)	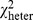	P value
		Latanoprost	Timolol			
Ocular						
Discomfort*	5	30/284	16/284	1.97(1.05,3.69)	0.82	0.03
Blurred vision	3	29/224	23/224	1.33(0.72,2.46)	3.70	0.37
Conjunctival hyperemia	4	29/254	12/254	2.72(1.33,5.59)	1.33	<0.01
Keratitis	1	9/137	8/138	1.14(0.43,3.05)	-	0.79
Uncontrolled IOP	2	4/59	8/58	0.48(0.14,1.62)	1.24	0.24
Total	7	129/343	103/342	1.49(1.05,2.10)	9.75	0.02
Systemic						
Headache	2	2/153	4/154	0.55(0.11,2.63)	0.16	0.45
Cardiac disorder**	2	0/96	6/94	0.13(0.02,1.08)	0.06	0.06
Dizziness	1	1/137	4/138	0.25(0.03,2.23)	-	0.21
Total	4	18/249	36/248	0.46(0.25,0.84)	1.41	0.01

* Discomfort include: eye discomfort, foreign body sensation, eye irritation.

**Cardiac disorder include: palpitation, cardiac arrhythmia.

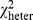
 = 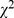 test for subgroup differences.

All pooling was undertaken using fixed effect model as no heterogeneity was detected by Q test.

1. **Ocular Adverse Effects.** Latanoprost caused more ocular adverse effects (OR = 1.49, 95% CI, 1.05∼2.10, P = 0.02) than timolol. The risks for discomfort and conjunctival hyperemia were significantly higher in latanoprost than timolol (P = 0.03 and P<0.01) (Table 4).
2. **Systemic Adverse Events.** More systemic adverse effects were observed in patients treated with timolol than latanoprost (OR = 0.46, 95% CI, 0.25∼0.84, P = 0.01). The risk for cardiac disorder was higher in timolol with a borderline statistical value (P = 0.06) (Table 4).

## Discussion

In this meta-analysis, the findings from 7 RCTs showed that latanoprost once daily could achieve lower IOP in Asian patients with CACG than timolol twice daily, with differences in absolute IOP reduction for mean, peak and trough of 2.3 mmHg, 2.4 mmHg and 2.5 mmHg, and differences in relative IOP reductions of 9.0%, 9.7% and 10.8%, respectively. These differences could last from 1 week up to 6 months with magnitudes of 2.0 mmHg, 2.0 mmHg, 1.9 mmHg, 1.9 mmHg, 2.3 mmHg, and 2.2 mmHg at 1 week, 2 weeks, 4 weeks, 8 weeks, 12 weeks and 24 weeks, respectively. Sensitivity analysis based on study design and withdrawal rate didn't change the results. There were no statistically significant differences in lowering IOP between Chinese Mainland population and Asia-Pacific population (non-Chinese Mainland). As for safety, latanoprost caused more ocular adverse effects and less systemic adverse events than timolol. Our findings confirmed the results of previous trials that latanoprost might be a substitute for timolol in the treatment of patients with CACG.

The difference of mean IOP reduction between these two drugs (2.3 mmHg and 9.0%) in patients with CACG was similar to that in patients with POAG or OH [11], [12], for whom the mechanism of latanoprost in reducing IOP was mainly due to increase uveoscleral outflow. One important inclusion criteria for the 7 trials was that there should be at least one quadrant without peripheral anterior synechiae. This criterion was a consideration of the traditional mechanism of latanoprost which was thought to be dependent on the area of visible ciliary body face. However, Ritch et al. [31] found that the efficacy of latanoprost in lowering IOP was independent of the height of ciliary body face. The trial by Kook et al. [32] demonstrated that latanoprost could still significantly reduce the IOP of CACG patients with no visible ciliary body face by percentages of 25.5%∼36.1%. Therefore, latanoprost might produce IOP-lowering effect via passage other than ciliary body face, for example, trabecular meshwork. If so, latanoprost might be a treatment choice for CACG patients at different stages, which deserved further study.

In this meta-analysis, it was found that both latanoprost and timolol were well tolerated by CACG patients. However, latanoprost produced more cases of ocular discomfort (P = 0.03) and conjunctival hyperemia (P<0.01) than timolol, but less systemic advents especially cardiac disorder (P = 0.06). As a whole, latanoprost produced higher risk of ocular adverse effects (P = 0.02) and lower risk of systemic adverse events than timolol (P = 0.01). More ocular adverse effects observed in patients treated by latanoprost might be related to its proinflammatory effect [33]. In addition, higher percentage of benzalkonium chloride, a known irritant of conjunctiva and cornea [34], was involved in latanoprost eyedrop (0.2 mg/ml) than that in timolol eyedrop (0.1 mg/ml), which might be another reason of ocular discomfort [25]. On the contrary, it should be noted that systemic adverse events were more serious than ocular adverse effects. One trial [30] included in the meta-analysis reported that some cases from the timolol group had to stop using timolol because of its systemic adverse events. In view of this, latanoprost may be more suitable for those CACG patients who had concurrently systemic diseases.

The seven trials included in the present study had relatively high quality (Figure 2). Most trials suffered no clear bias in randomization, allocation concealment and outcome reporting. Although only three of these seven trials were free of bias in terms of blinding, sensitivity analysis based on blinding found no significant difference. Considering that the effect of eyedrops is usually dependent on the compliance of patients, we also did sensitivity analysis based on withdraw rate less than 10% or not. There was no significant difference between the two subgroups. Although only seven trials were included, we did funnel plots and found no publication bias. From the above, we believe that the results in this meta-analysis are robust.

Besides the discriminating among mean, peak, and trough moments, the analysis included both absolute and relative IOP reduction. The included studies did not vary in concentration of the drug, moment of applying, and frequency of dosing for the different medicines. Latanoprost 0.005% eye drops were directly compared with timolol 0.5% eye drops in all of the trials. We undertook subgroup analysis and sensitivity analyses to explore the heterogeneity between population, study design and withdrawals and the results were all robust. However, it should be noted that the differences between these two drugs might be only due to the different frequency of use daily. That is, latanoprost once daily might produce better compliance than timolol twice daily [35], [36]. This issue needs to be addressed in future studies.

Some limitations remain in the present study. Firstly, only 7 studies met our inclusion criteria and were included in this meta-analysis, and the numbers of participants in the trials were also limited (range, 30∼275). Secondly, several trials lacked adequate randomization, allocation concealment, masking, and complete outcome data, which may leave them vulnerable to bias. The imputation of missing values also introduces bias. However, the studies that contributed the most weight to this meta-analysis were also the most methodologically stringent, and according to the result of sensitivity analysis, it is unlikely that poorer quality trials significantly biased the pooled estimates. Thirdly, timolol is commonly used in Europe as 0.25% slow release gel, once or twice daily, so the applicability of the present study may not be as relevant in other populations. Fourthly, not all included studies have all the data for all time points. This might cause bias to the results. Finally, publication bias is inevitable; our research was restricted to studies published in journals or in certain trial registers.

In summary, the present meta-analysis showed that latanoprost once daily could achieve better effect in lowering IOP of CACG patients than timolol twice daily, with higher risk of ocular adverse effects but lower risk of systemic adverse events. Thus, latanoprost may be a good substitute for timolol to lower IOP of CACG patients.

## Supporting Information

Figure S1 **Subgroup analysis of IOP reduction between latanoprost and timolol in studies with and without pharmaceutical industry funding.**
(TIF)

Table S1 **Search strategy and results.**
(DOCX)

Table S2 **Sensitivity analysis of absolute IOP reduction at peak between latanoprost and timolol.**
(DOCX)

Table S3 **Subgroup analysis of IOP reduction between latanoprost and timolol in Chinese Mainland population and Other Asia Pacific population.**
(DOCX)

Checklist S1 **PRISMA Checklist.**
(DOC)
