# Supplementary figures and images for: Meta-Analysis of Randomized Controlled Trials Comparing Latanoprost with Timolol in the Treatment of Asian Populations with Chronic Angle-Closure Glaucoma
Source: PLoS One. 2014 May 9;9(5):e96852. doi: 10.1371/journal.pone.0096852 (PMC4016135; doi:10.1371/journal.pone.0096852)

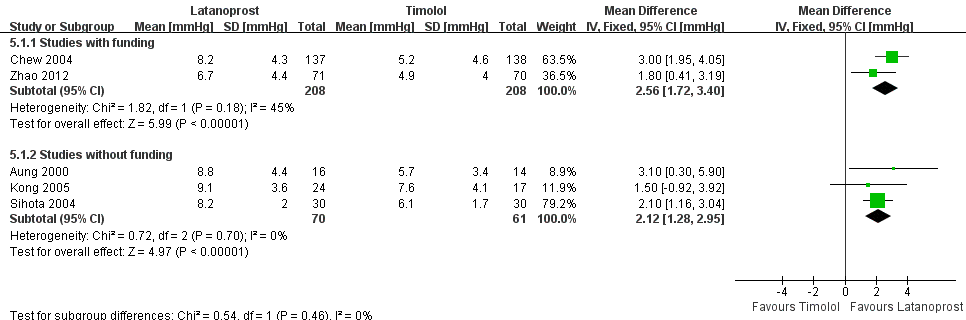

Supplement: Figure S1 — Subgroup analysis of IOP reduction between latanoprost and timolol in studies with and without pharmaceutical industry funding. (TIF) [file pone.0096852.s001.tif]
