# Supplementary material for: Meta-Analysis of Randomized Controlled Trials Comparing Latanoprost with Timolol in the Treatment of Asian Populations with Chronic Angle-Closure Glaucoma
Source: PLoS One. 2014 May 9;9(5):e96852. doi: 10.1371/journal.pone.0096852 (PMC4016135; doi:10.1371/journal.pone.0096852)
Supplement: Table S1 — Search strategy and results. (DOCX) [file pone.0096852.s002.docx]

**Table S1. Search strategy and results**

| No. | Search history | Results |
| --- | --- | --- |
| Stage 1 Diseases | | |
| 1 | "Glaucoma, Angle-Closure"[Mesh] | 2056 |
| Stage 2 Drugs | | |
| 2 | "latanoprost" [Supplementary Concept] | 1164 |
| 3 | “Xalatan” [Mesh] (same with 2) | 1164 |
| 4 | "Timolol"[Mesh] | 3119 |
| 5 | "Timoptol” [Mesh] (same with 4) | 3119 |
| 6 | 1 AND 2 AND 4 | 11 |
