# Supplementary material for: Meta-Analysis of Randomized Controlled Trials Comparing Latanoprost with Timolol in the Treatment of Asian Populations with Chronic Angle-Closure Glaucoma
Source: PLoS One. 2014 May 9;9(5):e96852. doi: 10.1371/journal.pone.0096852 (PMC4016135; doi:10.1371/journal.pone.0096852)
Supplement: Table S2 — Sensitivity analysis of absolute IOP reduction at peak between latanoprost and timolol. (DOCX) [file pone.0096852.s003.docx]

**Table S2. Sensitivity analysis of absolute IOP reduction at peak between latanoprost and timolol**

| Design & Withdrawals | No. of trails | No. of patients | | Mean difference in absolute IOP reduction (mmHg) (95%CI) | $\chi_{\text{subg}}^{2}$(p value) | Mean difference in relative IOP reduction(%)(95%CI) | $\chi_{\text{heter}}^{2}$(p value) |
| --- | --- | --- | --- | --- | --- | --- | --- |
|  |  | Latanoprost | Timolol |  |  |  |  |
| All trails | 6 | 266 | 256 | 2.4(1.9,2.9) |  | 9.7(7.6,11.8) |  |
| Design of trial | | | | | | |  |
| Double blind or single blind | 4 | 207 | 199 | 2.9(1.9,3.8) | 1.28(0.26) | 11.1(7.5,14.7) | 0.86(0.36) |
| Open label or not reported | 2 | 59 | 57 | 2.2(1.6,2.8) |  | 9.0(6.5,11.5) |  |
| Withdrawals | | | | | | |  |
| Withdrawals <10% | 4 | 213 | 212 | 3.0(2.2,3.9) | 2.68(0.10) | 11.4(8.0,13.3) | 1.53(0.22) |
| Withdrawals>10% | 2 | 53 | 44 | 2.1(1.5,2.7) |  | 8.7(6.2,11.3) |  |
| $\chi_{\text{subg}}^{2}$=$\chi^{2}$ test statistic for subgroup differences;$\chi_{\text{heter}}^{2}$=$\chi^{2}$ test for subgroup differences | | | | | | | |
| All pooling was undertaken using fixed effect model as no heterogeneity was detected by Q test | | | | | | | |
