# Supplementary material for: Meta-Analysis of Randomized Controlled Trials Comparing Latanoprost with Timolol in the Treatment of Asian Populations with Chronic Angle-Closure Glaucoma
Source: PLoS One. 2014 May 9;9(5):e96852. doi: 10.1371/journal.pone.0096852 (PMC4016135; doi:10.1371/journal.pone.0096852)
Supplement: Table S3 — Subgroup analysis of IOP reduction between latanoprost and timolol in Chinese Mainland population and Other Asia Pacific population. (DOCX) [file pone.0096852.s004.docx]

**Table S3. Subgroup analysis of IOP reduction between latanoprost and timolol in Chinese Mainland population and Other Asia Pacific population**

| Time Points | No. of trails | | Mean difference in IOP reduction (95%CI) | | | $\chi_{\text{subg}}^{2}$ (p value) | $\chi_{\text{heter}}^{2}$  (p value) |
| --- | --- | --- | --- | --- | --- | --- | --- |
|  | Chinese Mainland population | Other Asia Pacific population | Chinese Mainland population | Other Asia Pacific population | Total |  |  |
| Absolute IOP reduction (mmHg) | | | | | | | |
| Diurnal curve | 2 | 3 | 1.7(0.5,2.9) | 2.5(1.9,3.2) | 2.3(1.8,2.9) | 1.31(0.25) | 3.09(0.54) |
| Peak | 3 | 3 | 2.1(1.5,2.7) | 3.2(2.2,4.2) | 2.4(1.9,2.9) | 3.28(0.07) | 5.26(0.39) |
| Trough | 1 | 3 | 2.7(0.1,5.3) | 2.4(1.5,3.3) | 2.5(1.6,3.3) | 0.04(0.85) | 2.23(0.53) |
| Relative IOP reduction (%) | | | | | | | |
| Diurnal curve | 2 | 3 | 7.3(2.4,12.2) | 9.6(6.8,12.4) | 9.0(6.6,11.4) | 0.63(0.43) | 0.91(0.92) |
| Peak | 3 | 3 | 8.7(6.3,11.1) | 12.2(8.3,16.1) | 9.7(7.6,11.8) | 2.26(0.13) | 3.96(0.56) |
| Trough | 1 | 3 | 11.7(1.3,22.1) | 10.7(7.0,14.4) | 10.8(7.4,14.3) | 0.03(0.86) | 2.02(0.57) |
| $\chi_{\text{subg}}^{2}$=$\chi^{2}$ test statistic for subgroup differences;$\chi_{\text{heter}}^{2}$=$\chi^{2}$ test for subgroup differences | | | | | | | |
| All pooling was undertaken using fixed effect model as no heterogeneity was detected by Q test | | | | | | | |
